# Preparation and antitumor evaluation of hinokiflavone hybrid micelles with mitochondria targeted for lung adenocarcinoma treatment

**DOI:** 10.1080/10717544.2020.1748760

**Published:** 2020-04-06

**Authors:** Yuting Chen, Xue Feng, Luya Li, Kewei Song, Lantong Zhang

**Affiliations:** a Department of Pharmaceutical Analysis, School of Pharmacy, Hebei Medical University, Shijiazhuang, PR China;; b The Fourth Hospital of Shijiazhuang, Shijiazhuang, PR China

**Keywords:** Hinokiflavone, hybrid micelle, mitochondria-targeted, lung adenocarcinoma, nude mice

## Abstract

Hinokiflavone (HF) is a natural biflavonoid extracted from medicinal plants such as *Selaginella tamariscina* and *Platycladus orientalis*. HF plays a crucial role in the treatment of several cancers. However, its poor solubility, instability, and low bioavailability have limited its use. In this study, soluplus/d-α-tocopherol acid polyethylene glycol 1000 succinate (TPGS)/dequalinium (DQA) was applied to improve the solubilization efficiency and stability of HF. HF hybrid micelles were prepared via thin-film hydration method. The physicochemical properties of micelles, including particle size, zeta potential, encapsulation efficiency, drug loading, CMC value, and stability were investigated. The *in vitro* cytotoxicity assay showed that the cytotoxicity of the HF hybrid micelles was higher than that of free HF. In addition, the HF hybrid micelles improved anticancer efficacy and induced mitochondria-mediated apoptosis, which is associated with the high levels of ROS inducing decreased mitochondrial membrane potential, promoting apoptosis of tumor cells. Furthermore, *in vivo* tumor suppression, smaller tumor volume and increased expression of pro-apoptotic proteins were found in nude mice treated with HF hybrid micelles, suggesting that HF hybrid micelles had stronger tumor suppressive activity compared with free HF. In summary, HF hybrid micelles developed in this study enhanced antitumor effect, which may be a potential drug delivery system for the treatment of lung adenocarcinoma.

## 1. Introduction

Lung cancer is one of the malignant tumors with high metastasis and mortality in the world (Khan et al., 2016), of which non-small cell lung cancer (NSCLC) accounts for 85–90% (Hirsch et al., 2016). While lung adenocarcinoma is a type of NSCLC, chemotherapy plays a vital role in clinical anticancer treatment (Pilkington et al., 2015). Nevertheless, traditional chemotherapy drugs are limited by low bioavailability and non-specific targeting, which are prone to systemic unspecific toxicity. Some mitochondrial targeted drug delivery systems have been designed to solve the problem.

Mitochondria are important organelles that provide energy, and mitochondrial dysfunction is always associated with cell damage and apoptosis. The mitochondrial membrane potential in tumor cells is approximately –180 mV, which is much higher than that of normal epithelial cells, conducive to the accumulation of lipophilic cations in mitochondrial matrix (Agrawal et al., 2015; Tan et al., 2018). Thus, some lipophilic cations, such as triphenyl-phosphonium cation (TPP) and dequalinium (DQA) were modified onto the carriers (Wang et al., 2011b; Xu et al., 2017). Therefore, mitochondrial-targeted drug delivery system is an effective strategy for better tumor therapy.

Hinokiflavone (HF) is a natural biflavonoid extracted from medicinal plants such as *Selaginella tamariscina* and *Platycladus orientalis* (Zhang et al., 2011; Shan et al., 2018), possessing antitumor activities in KB human oral cancer and melanoma carcinoma cell lines (Shim et al., 2018; Yang et al., 2018). However, its poor solubility, instability and low bioavailability have limited its use (Chen et al., 2019). Therefore, it is necessary to select a suitable nanodrug delivery system to improve the solubility and bioavailability of HF.

Soluplus, polyvinyl caprolactam-polyvinyl acetate-polyethylene glycol, is an amphiphilic graft copolymer with the potential to enhance the solubility of hydrophobic drugs (Shamma & Basha, 2013; Hu et al., 2017). The polymer could form a micellar structure in a solution having a low critical micelle concentration (CMC), which has high dilution stability. In terms of molecular structure, soluplus has the hydrophilic portion of polyethylene glycol backbone and the hydrophobic portion of ethylene caprolactam/vinyl acetate side chain (Bernabeu et al., 2016).

d-α-Tocopherol acid polyethylene glycol 1000 succinate (TPGS), the derivative of natural vitamin E, is a nonionic surfactant with hydrophilic (PEG) head and a lipophilic (phytyl) tail (Mi et al., 2011; Guo et al., 2013). It could improve the drug solubility and drug encapsulation efficiency (EE). In addition, TPGS is capable of effectively inhibiting the efflux of some P-glycoprotein (P-gp) substrates, so as to overcome the multidrug resistance, and furthermore result in promoting the oral absorption and bioavailability of anticancer drugs (Collnot et al., 2006).

Dequalinium is an amphiphilic cationic surfactant, which is a synthetic quinoline derivative containing two isolated quinoline aromatic heterocycles. It has antimicrobial activity and is widely used to disinfect wounds and treat oral ulcers. In recent studies, DQA, as a compound with double positive charge, could selectively aggregate in the mitochondria of tumor cells due to the transmembrane potential (Shieh et al., 2011; Zupancic et al., 2014). The addition of cationic DQA to the hybrid micelle may enhance the electrostatic interaction between the micelles and the negatively charged membrane of cancer cells, thus promoting the absorption of the hybrid micelles by cancer cells (Dian et al., 2018).

In this study, we designed hybrid micelles of HF that could target mitochondria. The hybrid micellar formulation comprised of soluplus, TPGS, and DQA. The objective of this study is to characterize the mitochondrial targeting HF hybrid micelles, evaluate its cytotoxicity to lung adenocarcinoma cells *in vitro*, and study its anticancer effect in lung adenocarcinoma-bearing animals, and reveal its mechanism of action.

## 2. Materials and methods

### 2.1. Materials and instruments

Soluplus was kindly provided by BASF Auxiliary Chem. Co., Ltd. (Shanghai, China). Vitamin E polyethylene glycol succinate (TPGS) was purchased from Shanghai Yuanye Bio-Technology Co., Ltd. (Shanghai, China). Dequalinium was obtained from Shanghai Tao Shu Biotechnology Co., Ltd. (Shanghai, China). Hinokiflavone (purity >97%, 19202-36-9) was purchased from Chengdu Herbpurify Co., Ltd. (Chengdu, China). CCK-8 was purchased from Beijing Zoman Biotechnology Co., Ltd. (Beijing, China). Pyrene was obtained from Tixiai Chemical Industrial Development Co., Ltd. (Shanghai, China). Reactive oxygen species (ROS) assay kit, mitochondrial membrane potential detection kit, hematoxylin, goat serum, and DAB coloring solution were purchased from Beijing Solarbio Technology Co., Ltd. (Beijing, China). HE staining (hematoxylin–eosin) kit, bax, bcl-2 and caspase-3 were all purchased from Shenyang Wan Lei Biotechnology Co., Ltd. (Shenyang, China).

EYELLA N1100 rotary evaporator was obtained from Tokyo Rikakikai Co., Ltd. (Tokyo, Japan). Ultrasonic crushing instrument was obtained from Wuxi Worxin Instrument Manufacturing Co., Ltd. (Wuxi, China). Nano particle size tester was obtained from Malvern (Malvern, UK). Ultimate 3000 high performance liquid chromatography was obtained from Thermo Fisher Scientific (Waltham, MA). Dual function water bath thermostatic oscillator was obtained from Jiangsu Jintan Yitong Electronics Co., Ltd. (Changzhou, China). HF 240 cell incubator was obtained from Shanghai Li Shen Scientific Instrument Co., Ltd. (Shanghai, China). F 2500 fluorescence spectrophotometer was obtained from Hitachi High-Tech Company (Tokyo, Japan).

### 2.2. Preparation of HF hybrid micelles

HF hybrid micelles were prepared via thin-film hydration method. Briefly, 2 mg of HF, 64 mg of soluplus, 16 mg of TPGS, and 2 mg of DQA were co-dissolved in 30 mL of acetone. The solution was completely dissolved by ultrasound for 20 min, and the organic solvent was removed by rotary evaporator to form a thin film. Then, 8 mL of deionized water was added to the pear-shaped flask and ultrasound for 1 h at 25 °C. The HF hybrid micelle solution was filtered through a 0.22 μm syringe filter and the micelles were successfully prepared.

### 2.3. Characterization of HF hybrid micelles

Particle size, polydispersity index (PDI), and zeta potential of HF hybrid micelles were measured by Nano-ZS particle size analyzer. Each sample was evaluated three times and got the averaged results.

### 2.4. Micelle stability

In order to study the stability of HF hybrid micelles, the average particle size was determined when the micelles were preserved at 4 °C for 1, 5, 10, 20, and 30 days.

### 2.5. Determination of encapsulation efficiency and drug loading (DL)

The concentration of HF was analyzed by high performance liquid chromatography. The chromatographic separation was carried on a C_18_ column (ZORBAX SB-C18, 5 μm, 4.6 mm × 150 mm, Agilent, Santa Clara, CA). The mobile phase consisted of water containing 0.1% formic acid (A) and acetonitrile (B). A gradient elution program was conducted as follows: 45–65% B from 0 to 10 min, 65–65% B from 10 to 15 min. In order to maintain the balance of column, the program should be adjusted to 45% B for 5 min before the next injection. The injection volume was set as 10 μL and the mobile phase flow rate was 1 mL/min. The detection wavelength was 330 nm.

The EE and DL were determined as follows. One milliliter of micelles was diluted with 9 mL of methanol, vortexed for 15 min and sonicated for 15 min to disrupt the core–shell structure of the micelle, so that HF was released from the micelles and then the solution was centrifuged at 12,000 rpm for 5 min. The supernatant was determined by HPLC and the injection was repeated three times. The EE and DL were calculated as follows:

$$\mathrm{EE}\%=\frac{\text{the weight of HF in nanomicelles}}{\text{the weight of HF added in nannomicelles preparation}}\times 100\%$$

$$\mathrm{DL}\%=\frac{\text{the weight of HF}}{\text{the weight of HF},\;\mathrm{soluplus},\;\mathrm{TPGS},\text{ and DQA}}\times 100\%$$

### 2.6. Critical micelle concentration

The CMC value is an important parameter to characterize the self-assembly characteristics and structural stability of the polymer. It is determined by the pyrene fluorescence method. A series of polymer solutions of 1 × 10^−5^ mg/mL to 0.5 mg/mL were mixed with 6 × 10^−7^ mol/L pyrene. Then homogenized for 2 h, and then immersed in a water bath shaker at 37 °C overnight (Li et al., 2018). The fluorescence of the samples was measured by F2500 fluorescence spectrometer with an emission spectrum of 350 nm and an excitation wavelength of 339 nm. The CMC was calculated by analyzing the fluorescence intensity ratio at 371 nm (*I*_371_) to 381 nm (*I*_381_) and the polymer concentration.

### 2.7. Cell cultures

Human lung adenocarcinoma A549 cells were grown in F-12 supplemented with 10% fetal bovine serum (FBS) and 1% antibiotics (penicillin 100 U/mL and streptomycin 100 μg/mL) in an incubator containing 5% CO_2_ at 37 °C.

### 2.8. Cytotoxicity

A549 cells were seeded at a density of 1 × 105 cells/well in 96-well culture plate. After 24 h, the medium was removed and then the cells were washed with PBS twice. Drug was added into 96-well culture plates, including various concentrations of free HF and HF hybrid micelles with fresh culture media. The concentration of HF ranged from 0.78 μg/mL to 50 μg/mL and six duplicate wells per concentration. The blank control experiments were added blank culture medium. The control experiments were added to blank culture medium and A549 cells. The cells were further incubated for 24 h, and all the solutions in the 96-well plate were discarded. Each well was washed three times with 100 μL of PBS, and 10 μL of CCK-8 solution was added to each well in the dark. After incubation for 2 h under the condition of 5% CO_2_ at 37 °C, the absorbance of each well at 450 nm was measured by a microplate reader, and each group was repeatedly measured three times. GraphPad Prism 5 was applied to calculate the IC_50_ value of free HF and the HF hybrid micelles (GraphPad Software, La Jolla, CA). Cell viability was calculated according to the following formula:

cell viability (%)=[(As−Ab)/(Ac−Ab)]×100%.

where As is the experimental well, Ac is the control well, and Ab is the blank well.

### 2.9. Mitochondrial targeting

#### 2.9.1. ROS assay

ROS levels were determined using DCFH-DA (Li et al., 2013). DCFH-DA could be hydrolyzed by intracellular esterase to form DCFH, and ROS could oxidize non-fluorescent DCFH to form fluorescent DCF (strong green fluorescent substance). The level of ROS could be known by detecting the fluorescence of the DCF. A549 cells were treated with free HF and HF hybrid nanomicelles. The concentration of HF was 20 μg/mL. After 24 h incubation, 1 mL DCFH-DA stock solution was added to each well. The cells were then incubated for 30 min in the dark. Fluorescence intensity was measured immediately using a FACScan flow cytometer.

#### 2.9.2. Mitochondrial depolarization

Mitochondrial membrane potential (ΔΨm) was measured using the cationic lipophilic fluorochrome JC-1 (Wang et al., 2011a). Mitochondria depolarization was measured by the conversion from the red to green fluorescence intensity. Briefly, A549 cells were seeded in a six-well culture plates at a density of 1 × 10^5^ cells/well. After 24 h, the cells were treated with free HF and HF hybrid nanomicelles under the condition of 5% CO_2_ at 37 °C. The final concentration of HF was 20 μg/mL. Control experiments were performed by adding blank medium. After 24 h, the cells were washed with PBS twice and incubated with 500 μL JC-1 for 20 min under the condition of 5% CO_2_ at 37 °C in dark. The cells were then washed with PBS three times and immediately analyzed by a FACScan flow cytometry (Shi et al., 2018). The green emitted fluorescence signals were used to measure the loss of ΔΨm.

### 2.10. *In vivo* tumor suppression

Four-week-old healthy female BALB/c nude mice were selected to evaluate the *in vivo* antitumor activity of the HF hybrid micelles. The nine nude mice were randomly divided into three groups of three rats per group: (A) control group, (B) free HF, and (C) HF hybrid micelles. All animal experimental procedures were performed in accordance with the guidelines of the Care and Use of Laboratory Animals and were approved by the Animal Experimental Ethics Committee of Hebei Medical University. Approximately, 5 × 10^6^ A549 cells were subcutaneously injected into the back of nude mice to establish the tumor models. The volume of tumor was observed daily from the day of inoculation. When the tumor volume of each group reached about 100 mm^3^, the day was designated as day 0 (Chen et al., 2013). From day 2, groups A, B, and C were intragastrically administered with saline, HF (80 mg/kg) and HF hybrid micelles (80 mg/kg), respectively. The time of oral administration was days 2, 4, 6, 8, 10, and 12. The tumor volume was obtained 1–12 days before 14 days by measuring the longest diameter and shortest diameter of the tumor with a vernier caliper, calculating the tumor volume, and plotting the tumor growth curve. The nude mice were sacrificed on the 14th day, and the tumor tissues were excised, weighed and frozen for subsequent experiments. The tumor volume and tumor inhibition rate were calculated from the formula:

$$\text{Tumor volume}=(L\times W^{2})/2$$

where *L* is the longest diameter and *W* is the shortest diameter perpendicular to the length.

$$\text{Tumor inhibition rate }(\%)=(W_{c}-W_{e})/W_{c}$$

where *W*_c_ is the weight of the tumor in the control group and *W*_e_ is the weight of the tumor in the experimental group.

### 2.11. H&E staining

The lung and tumor tissues that were harvested from pretreated mice of each group were fixed in xylene, cut into 5 μm slices, and stained with hematoxylin and eosin by HE staining.

### 2.12. Immunohistochemical analysis

To further verify whether the antitumor activity of HF hybrid micelles was attributed to the induction of apoptosis *in vivo*, immunohistochemical analysis was carried out on tissue sections of tumor in three groups.

The steps of immunohistochemistry were as follows. After deparaffinization in xylene, the tissue sections of tumor were re-hydrated in graded series of ethanol (95%, 85%, and 75%), washed under distilled water and clarified with PBS. The antigen retrieval solution is heated in a microwave until it boils. The section rack was put into the antigen retrieval solution and heated continuously for 10 min. Then, the slicing rack was taken out and soaked into PBS. The sections were incubated at 3% H_2_O_2_ for 15 min to eliminate the endogenous peroxidase activity, and then washed with PBS. The serum of normal goats was added dropwise, incubated at room temperature for 15 min, and the serum was decanted. Sections were incubated overnight at 4 °C in a sealed humidity chamber with the primary antibody for Bax, Bcl-2, and caspase-3 (1:100 dilution). The HRP-labeled secondary antibody was diluted 500-fold with PBS and added dropwise to completely cover the tissue, then incubated at 37 °C for 60 minutes and immersed in PBS for five minutes with three times. One hundred microliters of 3,31-diaminobenzidine (DAB) was added dropwise, and immediately placed in water to terminate the reaction when the color just became dark, then counterstained with hematoxylin. After dehydration and transparency, the tissue sections were fixed with neutral gum and analyzed by light microscopy (×400 magnification).

### 2.13. Statistical analysis

All data are expressed as the mean ± standard deviation (SD) of three independent measurements. Statistical analysis was assessed using one-way analysis of variance. The *p* value <.05 was considered statistically significant.

## 3. Results

### 3.1. Optimization of the preparation process of HF hybrid micelles

In this study, HF hybrid micelles were prepared by the thin-film hydration method. Important factors affecting the physiochemical properties of HF hybrid micelles included hydration time, hydration temperature, DQA dosage, TPGS/soluplus ratio, and drug/carrier ratio. A single factor test method was used to evaluate the effects of the above factors on HF hybrid micelles preparation. It could be concluded from Table 1 that the particle size of micelles has tended to be stable since the hydration time began at 60 min. When hydration time was too short, the particle size was larger, which might be because the micelle film was not completely dissolved in water and could not be evenly dispersed. In addition, the encapsulation rate was the highest at 60 min. Based on the above reasons, 60 min was finally selected as the hydration time. As shown in Table 2, with the increase of hydration temperature, the particle size of the micelles gradually increased and the EE gradually decreased, which might be because the higher hydration temperature has a certain influence on the stability of the drug. Based on the above consideration, the hydration temperature of 25 °C was selected. Table 3 shows that when DQA/HF ratio was 1:1 (w/w), the particle size was smaller, and the DQA dosage had little effect on the EE. Table 4 shows that when TPGS/soluplus ratio was 1:4, the micelles had the smallest particle size and the highest encapsulation rate. With the increase of TPGS, the particle size increased and the EE decreased, so the TPGS/soluplus ratio was 1:4. It could be concluded from Table 5 that when drug/carrier ratio was 1:41, the particle size was the smallest and the EE was also relatively stable. Too little total amount of carrier would cause incomplete drug encapsulation, while too much total amount of carrier would cause waste of carrier material, and the particle size and EE were not ideal. Therefore, the final choice of drug/carrier ratio was 1:41. In order to prepare HF hybrid micelles with the smallest particle size and the highest EE, we have optimized the preparation process to use a hydration time of 60 min, hydration temperature of 25 °C, DQA/HF ratio of 1:1 (w/w), TPGS/soluplus ratio of 1:4, and drug/carrier ratio of 1:41.

**Table 1.** Effect of different hydration time on micelles (*n* = 3).

Hydration time (min)	Particle size (nm)	Encapsulation efficiency (%)
20	74.58 ± 0.34	83.26 ± 0.37
40	70.93 ± 0.22	86.23 ± 0.12
60	65.85 ± 0.46	90.27 ± 0.63
80	64.79 ± 0.17	88.93 ± 0.22

**Table 2.** Effect of different hydration temperature on micelles (*n* = 3).

Hydration temperature (°C)	Particle size (nm)	Encapsulation efficiency (%)
25	66.73 ± 0.22	90.25 ± 0.13
45	73.28 ± 0.46	86.12 ± 0.24
65	75.84 ± 0.38	80.71 ± 0.37

**Table 3.** The effect of the amount of DQA on micelles (*n* = 3).

DQA/drug (w/w)	Particle size (nm)	Encapsulation efficiency (%)
1:2	69.31 ± 0.32	89.25 ± 0.28
1:1	65.97 ± 0.54	90.11 ± 0.25
2:1	72.18 ± 0.73	91.02 ± 0.14

**Table 4.** The effect of TPGS/soluplus ratio on micelles (*n* = 3).

TPGS/soluplus (w/w)	Particle size (nm)	Encapsulation efficiency (%)
1:4	65.82 ± 0.74	89.02 ± 0.43
2:3	68.53 ± 0.62	87.85 ± 0.36
3:2	70.26 ± 0.11	86.92 ± 0.57
4:1	71.95 ± 0.21	80.67 ± 0.31

**Table 5.** The effect of drug carrier ratio on micelles (*n* = 3).

Drug/carrier (w/w)	Particle size (nm)	Encapsulation efficiency (%)
1:31	82.74 ± 0.63	53.79 ± 0.21
1:41	67.83 ± 0.15	89.92 ± 0.07
1:51	72.46 ± 0.72	90.54 ± 0.13

### 3.2. Characterization of the micelles

**Figure 1.**
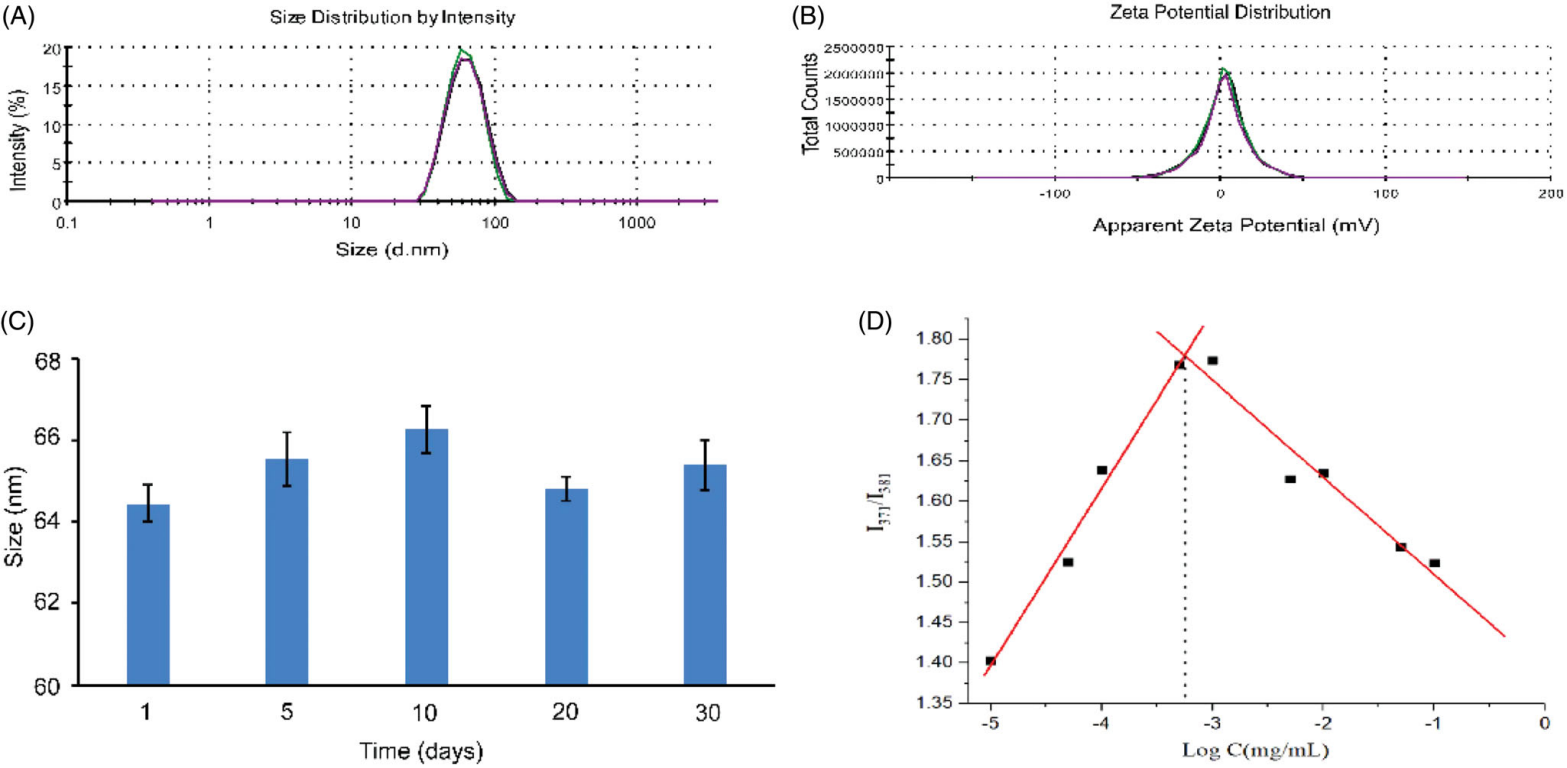
Characterization of HF hybrid micelles. (A) Size distribution spectrum of HF hybrid micelles; (B) zeta potential spectrum of HF hybrid micelles; (C) stability of HF hybrid micelles within 30 days; (D) measurement of the CMC of HF hybrid micelles.

As shown in Figure 1(A), the average particle size of HF hybrid micelles was 65.58 ± 0.66 nm, which had a narrow particle size distribution (PDI = 0.06 ± 0.01). Figure 1(B) shows that the zeta potential of HF hybrid micelles was 2.34 ± 0.21 mV. The EE and DL of HF hybrid micelles were 91.56%±0.73% and 2.23%±0.02%, respectively. As shown in Figure 1(C), the size of HF hybrid micelles changed slightly at 4 °C, and there was no precipitation within 30 days. With the increase of the copolymer concentration, when the micelles are formed, the fluorescence intensity of pyrene changed significantly. The CMC value of micelles was 5.55 × 10^−4^ mg/mL (Figure 1(D)), indicating that they had high stability and the ability to maintain their structure on dilution conditions.

### 3.3. Cytotoxicity

**Figure 2.**
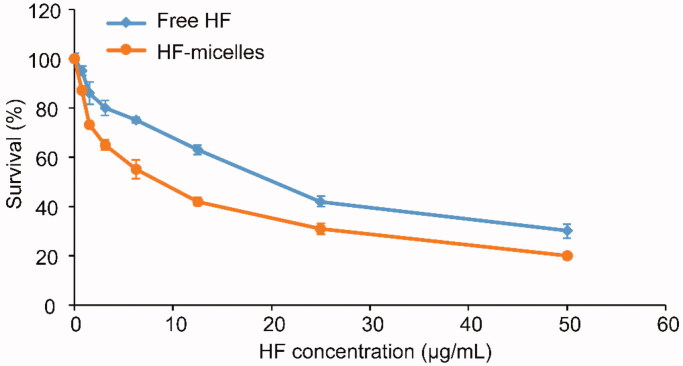
Survival rates of A549 cells after treatment with free HF and HF hybrid micelles.

CCK-8 assay was applied to measure the cytotoxicity of free HF and HF hybrid micelles. The results are shown in Figure 2 and the half maximal inhibitory concentration (IC_50_) of HF hybrid micelles at 24 hours (7.81 μg/mL) was lower than that of free HF (19.34 μg/mL), which was calculated by GraphPad Prism 5 (GraphPad Software, La Jolla, CA). Thus, the cytotoxicity of HF hybrid micelles was higher than free HF.

### 3.4. Mitochondrial targeting

#### 3.4.1. ROS assay

**Figure 3.**
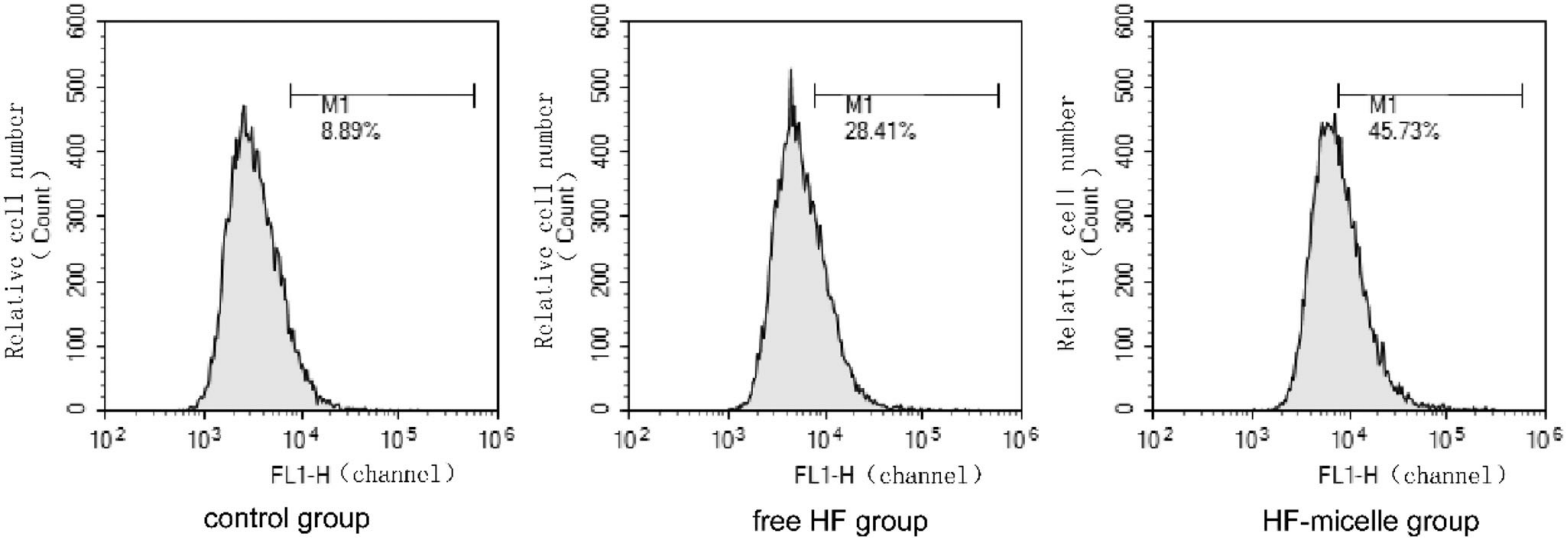
ROS levels determined by flow cytometry.

Mitochondrial damage could be associated with high levels of ROS. As shown in Figure 3, the value of M1 represented the proportion of cells labeled by the fluorescence probe DCFH-DA in the total cell samples, and the intracellular ROS could oxidize the non-fluorescent DCFH to generate fluorescent DCF. Hence, the level of ROS in the cells could be known by detecting the fluorescence of the DCF. It could be seen from the analysis of Figure 3, M1 was 45.73% after treatment with HF hybrid micelles, while M1 was 28.41% after treatment with free HF. Thus, ROS levels of the HF hybrid micelles group increased significant compared with that of HF.

#### 3.4.2. Mitochondrial depolarization

**Figure 4.**
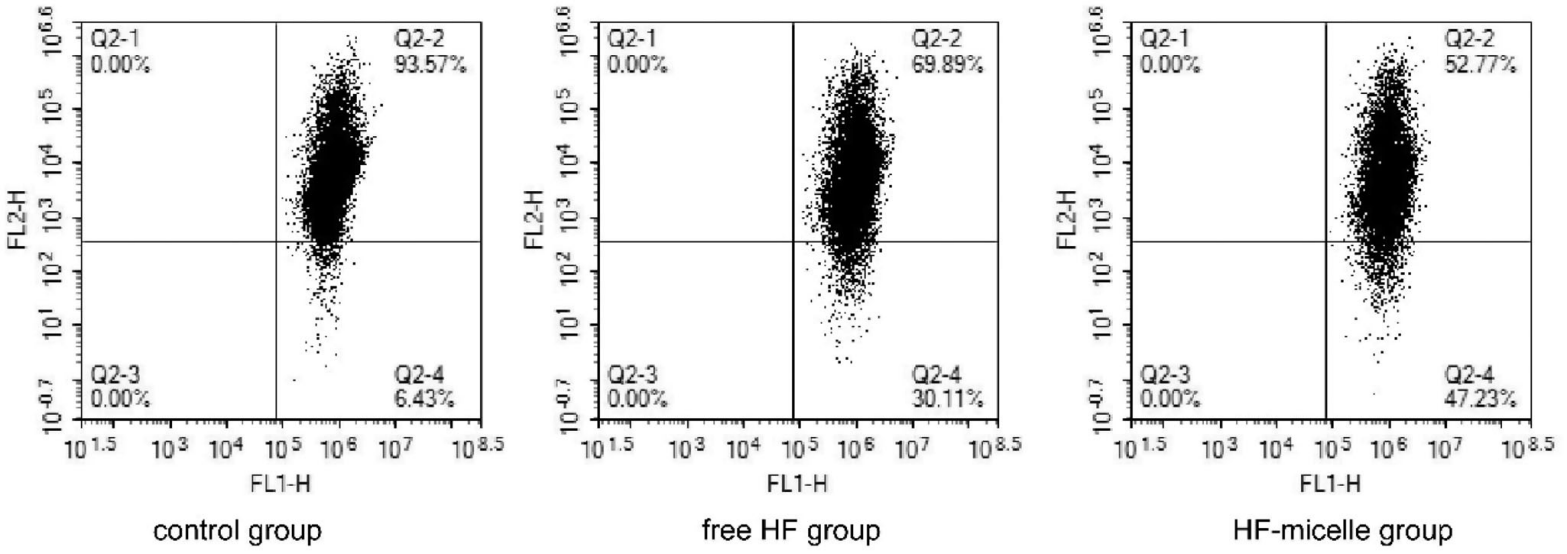
Mitochondrial membrane potential detection of free HF and HF hybrid micelles by flow cytometry.

ΔΨm was detected by flow cytometry using the JC-1 Detection Kit. The dye JC-1 could selectively enter mitochondria. In a highly polarized ΔΨm, JC-1 aggregated and glowed red. When ΔΨm depolarized, they form monomers and glowed green. When cells undergo apoptosis, JC-1 was released from the mitochondria, the red fluorescence intensity was weakened, and the green fluorescence intensity was enhanced. Thus, the transition of Q2-2 (normal cells) to Q2-4 (apoptotic cells) in Figure 4 was generated. As shown in Figure 4, the apoptosis rate was 30.11% after the treatment with free HF, and 47.23% after the treatment with HF hybrid micelles. As shown in Table 6, the ratio of JC-1 monomer/aggregate in HF hybrid micelle group was significantly increased, and the ratio of JC-1 monomer/aggregate in HF hybrid micelles was 2.08 times than that of the free HF, indicating that the mitochondrial membrane potential of the HF hybrid micelle group was significantly decreased compared with free HF, and the mitochondrial function was severely affected.

**Table 6.** JC-1 monomer/aggregate of different formulations compared with control group.

Group	Q2-1 (%)	Q2-2 (%)	Q2-3 (%)	Q2-4 (%)	JC-1 monomer/ aggregate (percentage of control)
Control group	0	93.57	0	6.43	–
Free HF group	0	69.89	0	30.11	6.27
HF hybrid micelle group	0	52.77	0	47.23	13.02

### 3.5. *In vivo* tumor suppression

**Figure 5.**
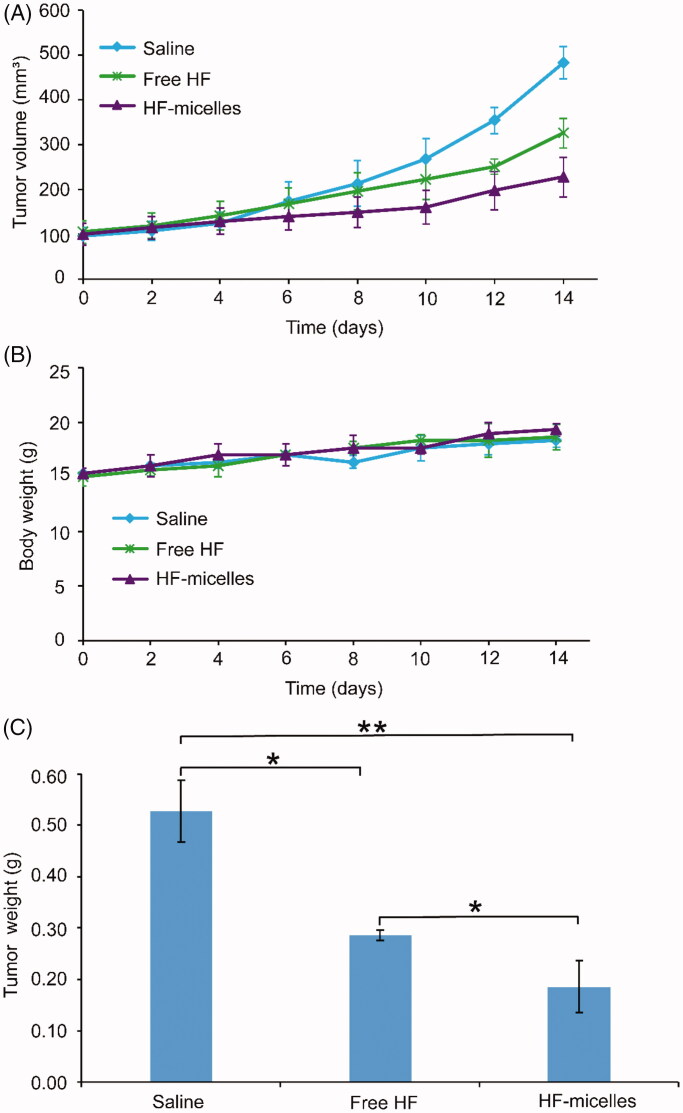
*In vivo* evaluation in A549 tumor xenografted nude mice model. (A) Tumor volume changes (*n* = 3). (B) Variations of body weight (*n* = 3). (C) Final tumor weights (mean ± SD, *n* = 3), *p<.05, **p<.01.

*In vivo* anti-tumor effects of HF hybrid micelles and free HF were studied in A549-xenografted nude mice. Figure 5(A) shows tumor growth curve after oral administration. The average tumor volume of the HF hybrid micelles group was significantly smaller than that of both the saline group (*p*<.01) and the free HF group (*p*<.05). As shown in Figure 5(B), the body weight of each experimental group increased to some extent, which was similar to the saline treated group. The final tumor weights are shown in Figure 5(C), indicating that the tumor weight of the HF hybrid micelles group was significantly lower than that of the normal saline group (*p*<.01) and the free HF group (*p*<.05). The HF hybrid micelles had the highest tumor inhibition efficiency (64.76%), which was significantly higher than free HF (45.92%).

### 3.6. H&E staining

**Figure 6.**
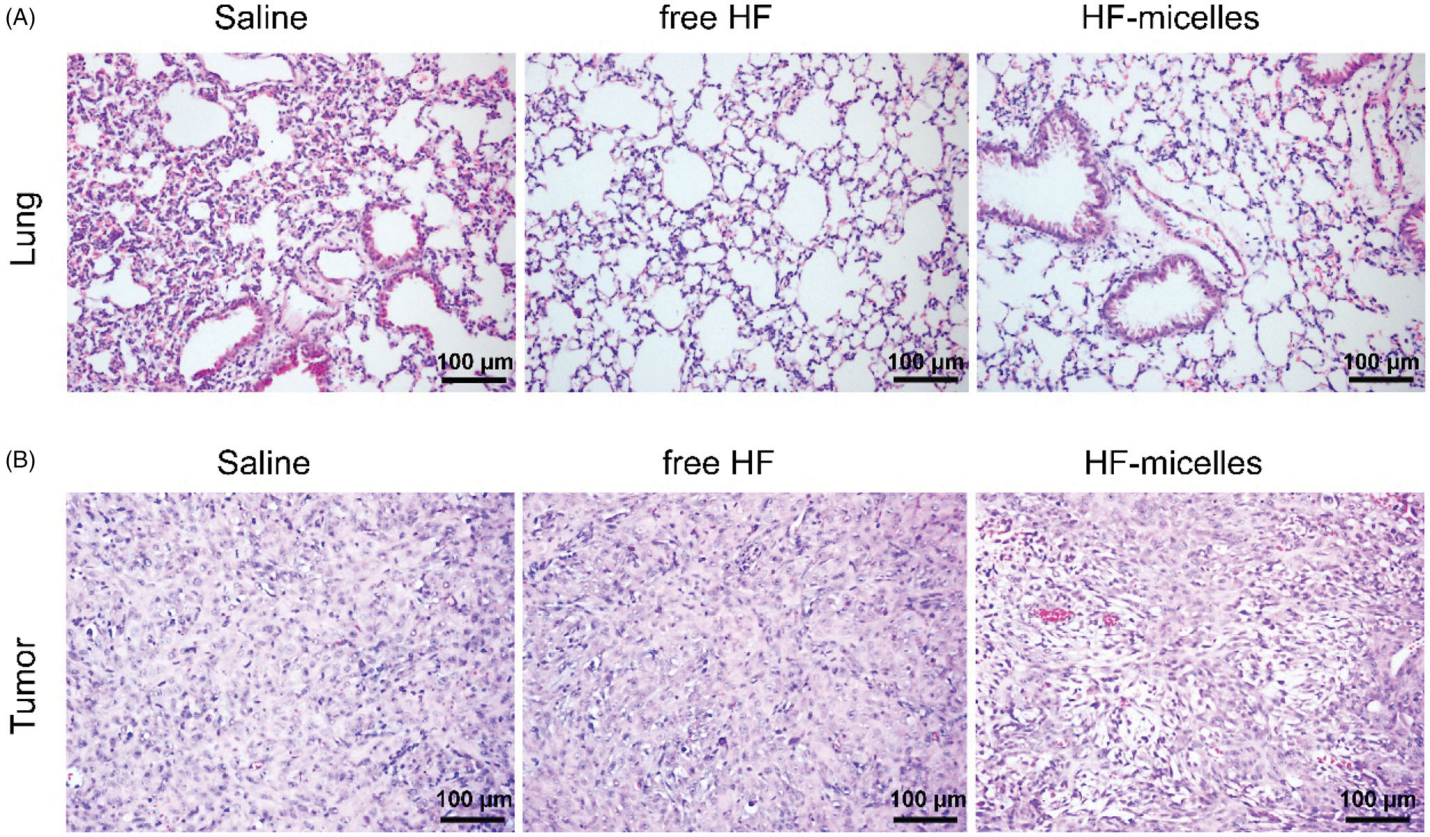
HE and Immunohistochemical images of three groups. (A) HE images of lung tissue in three groups. (B) HE images of tumor tissue in three groups.

As seen from HE images in Figure 6(A), the main organ section lung of the experimental group mice showed no obvious abnormality or lesions compared with the saline group mice. HE images in Figure 6(B) showed the pathological features of tumor tissues in three groups. After HF hybrid micelles treatment, lager numbers of nucleus of the tumor tissue were condensed compared with other groups, and large number of cells was necrotic.

### 3.7. Immunohistochemical analysis

**Figure 7.**
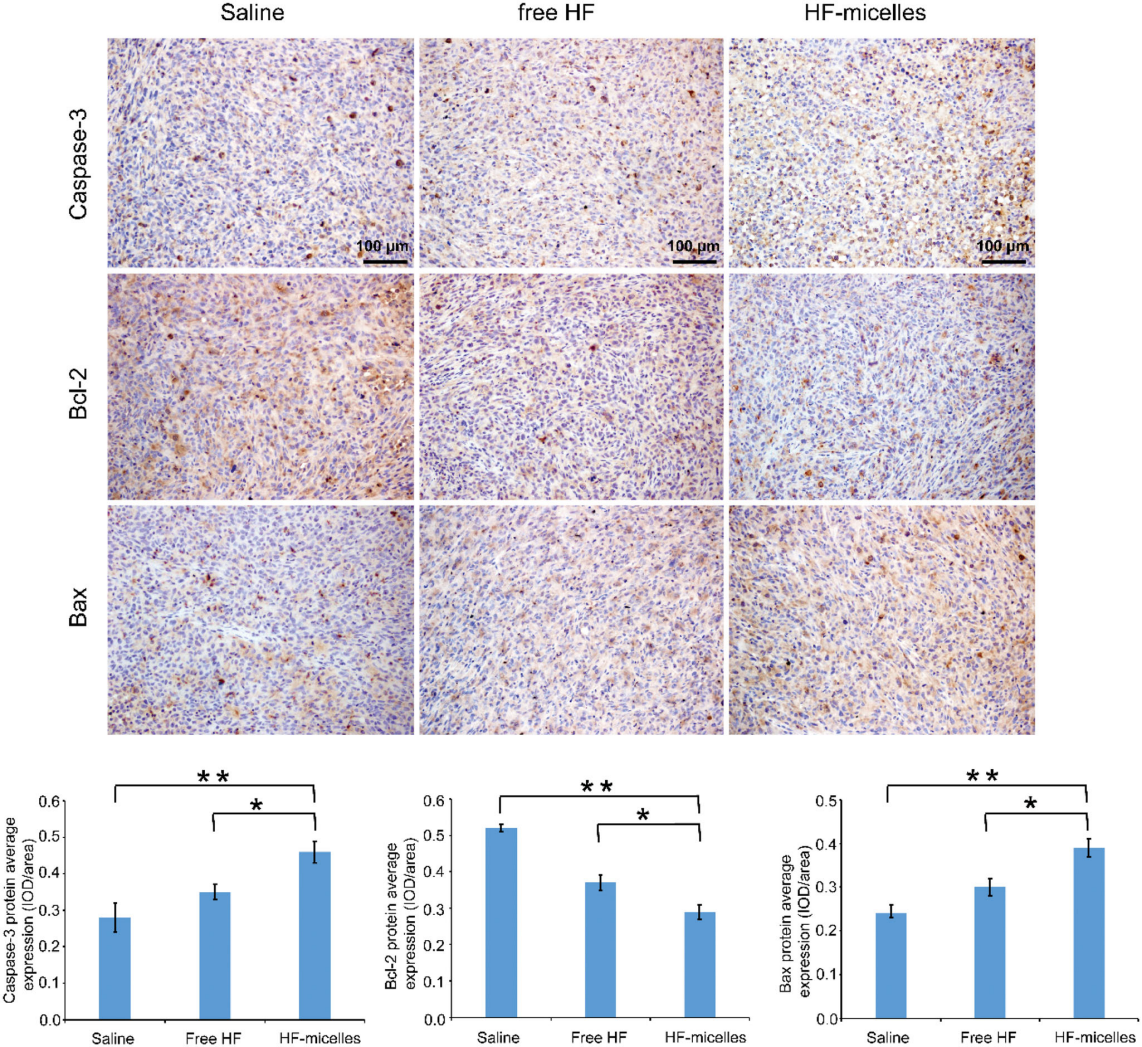
Immunohistochemical images of tumor tissue in three groups.

The relative expression levels of proteins in tumor tissues of different groups were detected by immunohistochemical staining. Image-Pro Plus 6.0 was used to quantitatively analyze immunohistochemistry. As shown in Figure 7, the average optical density of Bcl-2 expression in the HF hybrid micelles group was lower than that in the saline group (*p*<.01) and the free HF group (*p*<.05). The expressions of Bax and Caspase-3 proteins in the HF hybrid micelles group were higher than those in the saline group (*p*<.01) and the free HF group (*p*<.05). From the above, these results suggested that HF hybrid micelles enhanced the ratio of Bax/Bcl-2 and increased the expression of Caspase-3, accelerating tumor apoptosis.

## 4. Discussion

Nowadays, most chemotherapy drugs are administered intravenously, which are only fit for the treatment in hospitals. While the intravenous administration is inconvenient for patients who still need to continue chemotherapy at home after discharge, and the oral anticancer drugs will improve patient quality of life.

Natural products, such as paclitaxel, have been applied in the clinical treatment of cancers (Ashour et al., 2014; He et al., 2016). Due to the anticancer activity of HF, it has also attracted widespread attention. However, its poor solubility, stability, and low bioavailability limited its clinical application. In recent years, polymer micelles, liposomes, and nanoparticles have been used as drug carriers to overcome the disadvantages of drugs with low solubility. Hybrid micellar systems are considered to be one of the most promising strategies for improving the solubility and stability of hydrophobic drugs, which combine the outstanding advantages of different types of single polymer micelles (Zhao et al., 2017).

The constructed HF hybrid micelle had a small particle size (with a narrow distribution), high EE, and high stability. Micelles with particle size between 50 and 100 nm could increase oral absorption rate by promoting the gastrointestinal transport and absorbability of the blood system (Ramasamy et al., 2014, 2017). In addition, TPGS reduces the rapid uptake of micelles by the reticuloendothelial system, thereby increasing permeability and prolongation of drug retention time in the blood, furthermore, effectively accumulating the micelles to the tumor site. Moreover, the hydrophobic carbonyl group in the molecular structure of soluplus could form a hydrogen bond with the hydroxyl group of HF, thus increasing the DL and micellar stability.

*In vitro* cytotoxicity experiments showed that HF hybrid nanoparticles significantly increased the antitumor effect of A549 cells compared to free HF, which might be due to the mitochondrial targeting ability of HF hybrid micelles, which accelerated intracellular transport and mitochondrial accumulation of HF, and significantly promoted the increase in cytotoxicity. This mechanism could be explained in the following two aspects. On the one hand, TPGS inhibits drug efflux through the overexpressed ABC transporter on the cell membrane of cancer cells, thus promoting cell uptake (Wang et al., 2015). On the other hand, DQA-induced mitochondrial targeting effects increased apoptosis in A549 cells (Yu et al., 2012).

Mitochondria are the energy factories of cells, which are significant for the normal function of cells. The most important physiological function of mitochondria is the production of ATP by oxidative phosphorylation, but also to produce ROS, participate in cell apoptosis signal transduction, regulation of cytoplasm and mitochondrial calcium level. Mitochondrial damage is mainly associated with the increased amount of ROS in the mitochondria, and high levels of ROS can induce mitochondrial membrane potential decreased, promote apoptosis of tumor cells (Antonenko et al., 2008; Han et al., 2015; Yu et al., 2015). The results of mitochondrial depolarization and ROS assay showed that ROS levels in A549 cells were significantly increased after treatment with HF hybrid micelles compared with free HF. These results could be attributed to the combination of HF and mitochondrial target sites in the HF mixed micelles, which resulted in enhancing anti-tumor effects.

As for the *in vivo* tumor inhibition studies, the tumor volume and weight of HF hybrid micelles treatment were significantly reduced compared with free HF treatment, and control treatment, indicating that HF hybrid micelles had the highest tumor suppressive activity. In the mice weight change experiment, there was no significant difference among the three groups, indicating that HF hybrid micelles had no obvious toxicity. HE images of the main organ section lung suggested that the HF hybrid micelles had no obvious organ damage and further suggesting the HF hybrid micelles were safety drug delivery systems. HE images of tumor tissues in three groups indicated that HF hybrid micelle had high antitumor efficiency. To further study whether the antitumor efficiency of HF hybrid micelles was on account of inducing apoptosis *in vivo*, immunohistochemical staining in three groups of tumor tissue was performed to analyze the role of Bcl-2, Bax, and Caspase-3 in apoptosis. Thereinto, the ratio of Bcl-2/Bax reflects the extent of apoptosis. Bcl-2 was anti-apoptotic protein, while the proteins of Bax and Caspase-3 had pro-apoptotic effect (Wu et al., 2016). In summary, HF hybrid micelle treatment increased the ratio of Bax/Bcl-2, enhanced the expression of Caspase-3, and accelerated the tumor apoptosis. All these results indicate that the HF hybrid micelles had both strong tumor suppressor activity and little systemic toxicity.

## 5. Conclusions

In this study, we designed a hybrid micelle that encapsulated poorly soluble drug HF composed of soluplus, TPGS and DQA. The formulation with high EE had a particle size of about 65 nm and a low CMC value. For A549 cells, HF hybrid micelles showed higher antitumor effects than free HF *in vitro*. In addition, they effectively induce apoptosis of the cancer cells, which is related to the targeting of mitochondria by hybrid micelles. Moreover, HF hybrid micelles showed significant antitumor activity and little toxicity *in vivo*. The HF hybrid micelles developed in this study enhanced antitumor effect, which may be a potential drug delivery system for the treatment of lung adenocarcinoma.
